# Microplastics as both a driver of genitourinary cancers and a deliverer of treatments

**DOI:** 10.1038/s43856-026-01675-7

**Published:** 2026-05-27

**Authors:** Kannan Sridharan, Brigida Anna Maiorano, Farah Rehan, Francesca Maradonna, Elisabetta Giorgini, Tarek Taha, Javier Molina-Cerrillo, Sebastiano Buti, Francesco Piva, Francesco Massari, Matteo Santoni

**Affiliations:** 1 Department of Pharmacology & Therapeutics, College of Medicine & Health Sciences, Arabian Gulf University, Manama, Kingdom of Bahrain; 2 Department of Medical Oncology, IRCCS San Raffaele Hospital, Milan, Italy; 3 Department of Molecular Medicine, College of Medicine & Health Sciences, Arabian Gulf University, Manama, Kingdom of Bahrain; 4 Department of Life and Environmental Sciences, Università Politecnica delle Marche, Via Brecce Bianche, Ancona, Italy; 5 INBB-Consorzio Interuniversitario di Biosistemi e Biostrutture, Roma, Italy; 6 Royal Marsden NHS Foundation Trust, London, UK; 7 Department of Medical Oncology, Hospital Universitario Ramón y Cajal, Madrid, Spain; 8 Medical Oncology Unit, University Hospital of Parma, Parma, Italy; 9 Department of Medicine and Surgery, University of Parma, Parma, Italy; 10 Department of Specialistic Clinical and Odontostomatological Sciences, Polytechnic University of Marche, Ancona, Italy; 11 Medical Oncology, IRCCS Azienda Ospedaliero-Universitaria di Bologna, Bologna, Italy; 12 Department of Medical and Surgical Sciences (DIMEC), University of Bologna, Bologna, Italy; 13 Medical Oncology Unit, Macerata Hospital, Macerata, Italy; 14 Aron Research Foundation ETS, Macerata, Italy

**Keywords:** Renal cell carcinoma, Bladder cancer

## Abstract

Microplastics and nanoplastics (MNPs) are environmental contaminants increasingly detected in human tissues, raising public health concerns. Although evidence is still insufficient to directly link MNPs to genitourinary cancers (GU), this Review examines their potential role in prostate, bladder, and renal cell carcinoma. Proposed mechanisms include chronic inflammation, oxidative stress, genotoxicity, and endocrine disruption driven by plastic-associated additives. Emerging studies report quantitative detection of MNPs within human prostate and bladder tumors, with higher burdens associated with dietary habits such as frequent take-out food consumption. The Review also highlights their role in cancer therapy: MNPs may alter antineoplastic drug pharmacokinetics and promote resistance, yet polymer-based nanoparticles can be engineered as advanced drug delivery platforms. Materials such as PLGA and PEG may improve targeted delivery of chemotherapies and immunotherapies, supporting more effective and personalized treatment strategies in GU oncology.

## Abstract (web summary)

Sridharan et al. review the potential role of microplastics and nanoplastics in development of prostate, bladder and renal cell carcinoma. They also discuss how they can be used to deliver drugs to treat cancers.

## Introduction

Microplastics and nanoplastics (MNPs) are non-biodegradable solid particles composed of synthetic polymers, such as polypropylene (PP), polystyrene (PS), or polyethylene (PE), and chemical additives. Microplastics typically range in size from 1 µm to 5 mm, whereas nanoplastics are smaller particles, with a size <1 µm. Primary MNPs are intentionally manufactured for commercial applications (e.g., microbeads in cosmetics, biomedical products, soaps, and toothpaste). In contrast, secondary MNPs originate from the fragmentation of larger plastic due to environmental degradation and ultraviolet (UV) radiation^1^. The persistence of MNPs in the environment is exacerbated by their resistance to natural degradation and poor water solubility, leading to long-term accumulation in ecosystems^1^. Globally, plastic waste management remains inefficient, with 50% of plastics having a short lifespan, 30% an intermediate lifespan, and 20% enduring for extended periods. Due to high recycling costs, nearly 80% of plastic waste ends up in landfills or is indiscriminately released into the environment, further contributing to MNP pollution^2^. Once released, MNPs undergo physical and chemical transformations (e.g., UV-induced weathering, adsorption of pollutants) altering their mechanical properties and increasing their potential to interact with biological systems, posing significant risks to both environmental and human health^3^. Their ubiquitous presence has led to widespread contamination of air, water, soil, and the food chain, with documented accidental ingestion across diverse species, including fish, marine worms, seabirds, and ultimately, humans^4^. According to World Health Organization (WHO) guidelines, the safe limit for human exposure to styrene monomers is set at a time-weighted average of 20 parts per million (85 mg/m³), with a maximum short-term exposure of 40 ppm (170 mg/m³)^5^. Furthermore, a range of other hazardous substances used in plastic production are now pervasive environmental pollutants. These include catalysts and monofunctional peroxides (e.g., zeolites and iron (III) oxides), along with stabilizers and emulsifiers such as bis(2,2,6,6-tetramethylpiperidin-4-yl) decanedioate, which are all involved in creating polystyrene particles. Additionally, chemical initiators such as benzoyl peroxide and azobisisobutyronitrile, employed to speed up the polymerization process, are also found globally as environmental contaminants^6,7^.

**Fig. 1:**
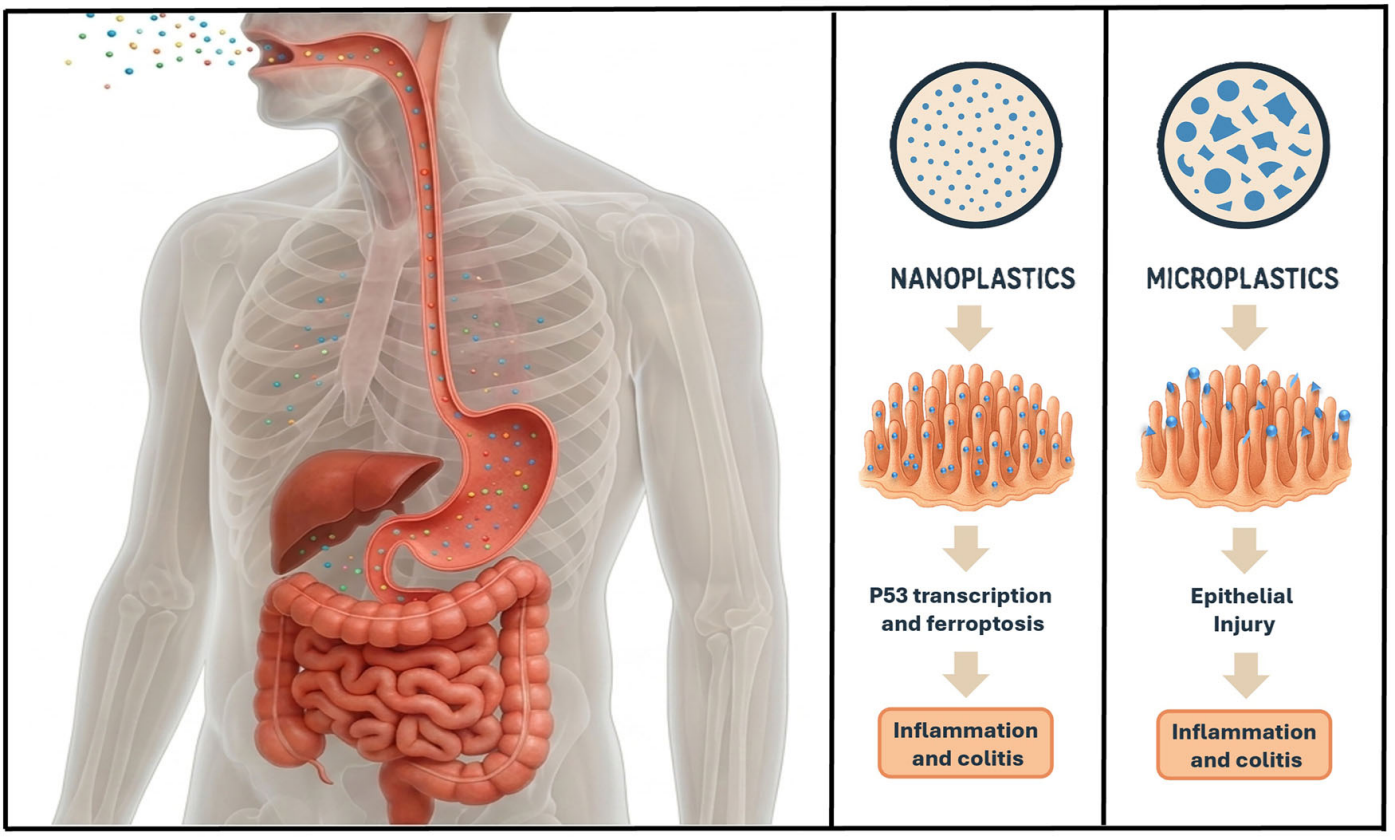
Size-dependent mechanisms by which MNPs cause intestinal epithelial injury. Adapted from [Cheng, Y., Chen, J., Fu, R. et al. Molecular mechanism differences between nanoplastics and microplastics in colon toxicity: nanoplastics induce ferroptosis-mediated immunogenic cell death, while microplastics cause cell metabolic reprogramming].

MNPs could be considered exposure biomarkers because they can be detected analytically, may persist over time, and capture contact with contaminated environments via ingestion, inhalation, or potentially dermal uptake. At the same time, substantial between-person variability, driven by differences in diet, exposure patterns, microbiome profiles, and physiological determinants of translocation and clearance, can produce highly heterogeneous internal burdens. Human exposure to MNPs occurs through multiple pathways, with an estimated average intake of 4.1 µg per week, equivalent to consuming a credit card’s worth of plastic annually (or roughly 50 plastic bags per year)^8^. The primary routes of exposure include enteral ingestion via contaminated food and water as shown in Fig. 1, with the highest intake from fruits and vegetables (4.78–5.58 × 10⁵ particles/year), drinking water (2.2–12 × 10⁵ particles/year), and table salt (5–7 × 10³ particles/year), and inhalation of airborne microplastics, particularly in indoor environments (1.6–2.3 × 10⁵ particles/year) compared to outdoor air (0.46–2.1 × 10⁵ particles/year)^9^. Dermal absorption, although less characterized, occurs through personal care products (e.g., soaps, hand washes, sunscreens, and toothpaste), the exact quantity absorbed remain unknown^10^. In 2019, the WHO reported no safety concerns for human health due to the presence of MNPs in drinking water^11^. However, since then, a growing body of evidence has investigated the potential harm of MNPs to various aspects of human health, including cancer. Indeed, MNPs are under investigation for their potential role in the development of various solid tumors, as they are involved in shaping the immune system and tumor microenvironment (TME), as well as in cell growth and proliferation^12^. There is limited evidence regarding the involvement of MNPs in the development of tumors in the genitourinary (GU) tract. However, this group of tumors has a widespread diffusion, with prostate cancer (PCa) being one of the most common cancers in the male population. Moreover, GU tumors are associated with excretory function, which is ultimately involved in metabolizing and eliminating various particles from the human body.

In this Review, we provide an overview of the relationship between MNPs and GU tumors, summarizing the link between MNPs and tumor development. Given their ready take up by the body we also discuss their interaction with cancer therapy, and potential future applications as antineoplastic therapeutic agents.

### The fundamental differences between environmental MNPs and engineered polymeric nanoparticles

A rigorous distinction should be made between unintentionally generated environmental micro-/nanoplastics (MNPs) and engineered polymeric nanoparticles designed for biomedical use, since they differ fundamentally in origin, degree of physicochemical control, exposure scenarios, and the strength of the safety evidence available (Table 1). From a physicochemical standpoint, environmental MNPs are intrinsically heterogeneous systems, characterized by mixed polymer composition, variable additive content, broad and polydisperse size distributions extending from the nanoscale to the millimeter scale, and irregular morphologies including fibers, fragments, films, and occasional spheres. Their surface properties are also highly dynamic: UV-driven oxidation, mechanical abrasion, and environmental weathering can modify roughness, hydrophobicity, dispersibility, and surface reactivity, while adsorbed biomolecules, biofilms, metals, and persistent organic pollutants further reshape the particle–biointerface in ways that are difficult to predict experimentally^13,14^. As a result, environmental MNPs typically display unstable and poorly standardized surface chemistry, charge, and contaminant burden, all of which can substantially influence biological interactions, tissue distribution, and toxicological readouts. Human exposure is typically chronic, involuntary, and occurs through multiple routes, most notably ingestion and inhalation. At the same time, estimating internal dose remains difficult because analytical approaches are heterogeneous and prone to contamination artifacts. Even so, biomonitoring investigations and scoping reviews have reported plastic particle detection in blood and several tissues, reinforcing the need for harmonized methodologies and shared quality criteria^15,16^. By contrast, polymeric nanoparticles used for drug delivery (e.g., PLGA- and PEG-based platforms and polymeric micelles) are deliberately manufactured with defined composition, tight size distributions, and adjustable surface features (such as PEGylation and targeting ligands), enabling more reproducible pharmacokinetics and biodistribution. PLGA carriers, in particular, are extensively studied because PLGA is a biodegradable polymer with longstanding use in pharmaceutical technologies; multiple reviews stress that successful clinical translation depends on standardized toxicology packages, along with stringent control of critical quality attributes, including composition, residual solvents, endotoxin burden/sterility, stability, and release kinetics^17,18^. Notably, PEGylation, while commonly employed to extend circulation time, also carries clinically meaningful considerations (e.g., anti-PEG antibodies and hypersensitivity reactions). These issues are typically addressed within controlled development programs and regulatory evaluations, rather than representing an uncontrolled environmental modifier of hazard or exposure^19,20^.

**Table 1:** Key differences between environmental MNPs and engineered nanoparticles

Property	Environmental MNPs (unintentional)	Engineered polymeric nanoparticles (intentional)
Origin^13^	Fragmentation/abrasion; secondary particles; mixed sources; environmental aging	Designed manufacturing for defined function
Composition^13–15^	Mixed polymers + additives; weathered/oxidized surfaces; adsorption of co-pollutants	Defined polymer/excipients; controlled residuals and degradation (e.g., PLGA hydrolysis); PEGylation as design feature
Size range & distribution^15^	Broad (nm–mm) and poorly standardized across studies	Typically narrow distributions (often tens–hundreds of nm)
Physicochemical predictability^13–15,19^	Low, due to aging, heterogeneity, and contaminant adsorption	High, due to controlled synthesis and quality-by-design manufacturing
Morphology^15^	Heterogeneous (fibers/fragments/films/spheres), irregular surfaces	Reproducible morphology (often spherical); controlled structure
Surface control^13,19^	Uncontrolled; oxidized/weathered; variable corona/biofilm; changing charge/hydrophilicity	Tunable (PEGylation, ligands, charge); reproducible
Contaminants burden^13,14,17^	High likelihood of adsorbed POPs/metals/PAHs + environmental bioburden	Low/controlled impurities; sterility/endotoxin testing in translational pipelines
Exposure context^15,17^	Chronic, involuntary; ingestion/inhalation; dose uncertain	Defined dose/route/schedule; toxicity monitoring
Evidence base^17^	Emerging; high heterogeneity; contamination bias; limited causal inference	Standardized preclinical packages; quality attributes/QbD emphasized for translation
Regulation^17,19^	Environmental pollutant; not regulated as a medicinal product	Regulated as drug/biologic/device depending on product; GMP/QC requirements
Intended function^19^	No intended biological function; potential hazard	Therapeutic function by design (delivery, targeting, controlled release)

### Regulatory landscape and translational implications

A key obstacle to effective translation into policy and practice is the continuing legislative and regulatory shortfall, which in many ways reflects the scientific fragmentation of the field. Reported levels of microplastic contamination are often not comparable across geographic areas because sampling, detection, and analytical workflows are not harmonized; with a lack of alignment among national and international legal frameworks representing a structural barrier to coherent, impactful public policy^21^. From a regulatory perspective, existing instruments frequently capture only part of the overall exposure problem. For instance, the European Union has implemented a REACH restriction aimed at reducing releases of synthetic polymer microparticles when they are intentionally present as such or deliberately incorporated into mixtures (e.g., certain consumer and industrial products). While this measure is relevant for primary microplastics, it does not directly address the larger contribution from secondary microplastics generated by the breakdown of macroplastics and by diffuse sources such as tire and road wear, textile shedding, and other forms of abrasion and fragmentation [ECHA microplastics^22,23^. In parallel, global governance remains in flux: negotiations toward a legally binding international instrument on plastic pollution are ongoing within UNEP’s Intergovernmental Negotiating Committee, underscoring that a fully coordinated global approach is still being constructed^24^.

Even though causal inference in humans remains incomplete, the regulatory gap has practical clinical relevance: in the absence of enforceable, harmonized exposure limits and routine standardized monitoring, prevention largely depends on upstream actions (product requirements, improved waste handling, and emission controls) rather than on clinical interventions. Thus narrowing legislative inconsistencies and standardizing measurement are best viewed as prerequisites for credible risk assessment and for any future clinical recommendations^21^.

### Human internal exposure (body burden), heterogeneity, and analytical limitations

Human biomonitoring investigations have described plastic particles in several biological matrices, including blood, urine, and placental tissue, supporting the notion that internal exposure can occur. At present, however, there is no clinically validated metric that reliably captures an individual “body burden,” nor are biological reference values or thresholds available. As a result, existing data are insufficient to define human dose–response relationships or to propose carcinogenic risk cutoffs. For example, the first quantitative, mass-based assessment reporting plastics in human blood estimated an average summed concentration in the µg/mL range, but the cohort was small and cross-study comparisons remain difficult because protocols and reporting practices vary substantially^15,25^. Similarly, Raman-based studies have reported microplastics in urine, yet the evidence is still early-stage and highly dependent on analytical sensitivity and stringent contamination control^26^. Reports using Raman and FTIR approaches on placenta samples further illustrate strong method dependence: detection frequencies and the apparent size spectrum can shift markedly depending on whether workflows only interrogate particles larger than ~50 µm or, alternatively, apply higher-sensitivity Raman procedures that capture particles in the low-micrometer range^27–29^.

Considerable interindividual variability is also expected. Exposure profiles differ with diet, drinking-water source, indoor air and occupational environments, smoking, socioeconomic conditions, and co-exposures common in industrialized settings. Moreover, host-related factors, such as intestinal permeability, renal clearance, and inflammatory status, may modulate translocation and retention. Consequently, the detection of plastics in human specimens should be interpreted primarily as evidence of internal exposure and biological plausibility, rather than as proof of a uniform, clinically interpretable, or actionable “toxic load.”

A further barrier to translation is analytical bias driven by non-harmonized measurement pipelines. Vibrational spectroscopy (µFTIR and µRaman) can provide particle-resolved information (polymer identification, size, and morphology), but performance is strongly influenced by particle size and matrix effects. In practice, many FTIR imaging workflows have effective lower size limits around ~20 µm, whereas optimized µRaman/µFTIR methods can reach roughly ~1–5 µm; detection of smaller nanoplastics remains difficult and is often close to instrumental and procedural limits^30,31^. By contrast, pyrolysis-GC/MS offers robust, chemistry-specific, mass-based polymer quantification, yet it does not retain particle size or shape and therefore may not map cleanly onto particle-number metrics that could matter for barrier crossing and tissue distribution. Comparative work indicates that FTIR imaging and pyrolysis-GC/MS can yield divergent estimates depending on sample complexity and the units reported (counts versus mass)^32^.

Overall, the field still lacks: (i) standardized protocols with rigorous contamination prevention, recovery assessment, and transparent quality reporting; (ii) harmonized reporting conventions (mass versus particle counts, size-class binning, and clearly stated limit of detection (LOD) / Limit of Quantification (LOQ); and (iii) well-designed prospective studies linking internal exposure measures to clinical endpoints while adequately addressing confounding. Therefore, although mechanistic findings and biomonitoring results support biological plausibility, it is premature to define clinical thresholds or to propose risk-stratified recommendations. Moreover, although a range of analytical methods is available to identify and characterize micro- and nanoplastics a widely accepted “gold standard” has yet to be defined.

Evidence on where micro- and nanoplastics localize at the subcellular level is derived mainly from cell-culture experiments and animal studies. In these models, particles are most often reported within the cytoplasmic compartment, frequently sequestered in endosomal and lysosomal vesicles, while nuclear proximity or entry has been described only in some conditions and appears to depend on parameters such as particle size, surface charge, and surface functionalization^33–35^. Although these observations are consistent with the biological plausibility of MNP-driven genotoxic effects, they cannot yet be translated straightforwardly to human genitourinary tissues. Indeed, direct and reproducible demonstrations of subcellular MNP localization in human GU specimens are currently lacking, largely because of methodological constraints.

### Biocompatibility and toxic effects of micro-nanoplastics in the human body

Current evidence does not identify any specific lifestyle as definitively responsible for microplastic accumulation in the bladder or prostate. However, habits associated with higher overall exposure, such as frequent consumption of bottled water, heating food in plastic containers, regular intake of take-away food, and prolonged exposure to indoor dust and synthetic fibers, may plausibly increase the burden of microplastics in the urinary system. After entering the human body, MNPs tend to accumulate in various tissues due to their persistence and poor degradability^26,36,37^.

**Fig. 2:**
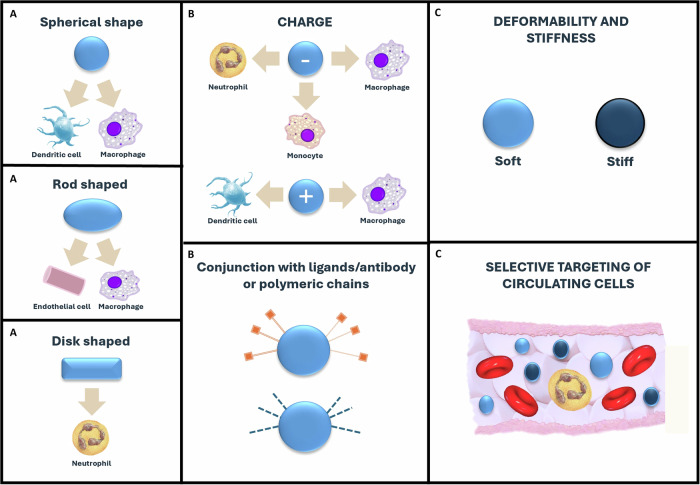
Optimization of nanoplastics carriers. **A** Nanoplastics can be formulated into a variety of shapes to target specific cell populations. **B** Functional groups on nanoplastics can be conjugated with targeting ligands or stealth polymeric chains. Additionally, nanoplastics can be positively or negatively charged. **C** The stiffness of the nanoplastics can be varied to target specific tumor cells in the bloodstream selectively.

The biocompatibility of MNPs, defined as their uptake, accumulation, and toxic effects in biological systems, is primarily determined by their size, shape, and surface properties (Fig. 2). The smaller the particle is, the more easily cells internalize it, resulting in higher accumulation rates^38^.

Experimental studies suggest that exposure to micro- and nanoplastics can elicit a broad range of biological effects, including disruption of epithelial and endothelial barrier integrity, hormone-linked alterations, and inflammatory as well as oxidative stress–related outcomes^39^. Here, we focus on outlining the overall toxicological pattern and the principal organ systems reported to be affected^9^. Moreover, because MNPs can adsorb toxic chemicals (e.g., heavy metals, persistent organic pollutants) on their surface, they may serve as carriers of additional harmful agents, amplifying their cytotoxic and genotoxic effects.

A scoping review of 129 studies highlighted potential links between MNP exposure and neurotoxicity, metabolic disorders, and an elevated risk of cancer, underscoring their emerging role as a public health concern^40^. After inhalation or oral intake, experimental research points to the lung and liver as key organs in which MNP-associated toxicity may manifest, and it also reports secondary changes affecting intestinal barrier integrity and the composition of the gut microbiota^41–43^. MNPs can also affect the reproductive system, with altered levels of testosterone, and reduced cell count and deformities in reproductive cells observed both in males and in females^44^.

### Micro-nanoplastics and cancer risk development

At the cellular level, microplastics enter cells via clathrin- and caveolae-mediated endocytosis, by modulating intracellular signaling networks and, in experimental systems, aligning with shifts indicative of aberrant cell growth and other features commonly associated with carcinogenic transformation^45^. Many plastic-associated chemicals, such as vinyl chloride, dioxins, phthalates, and benzene, are classified as known or probable carcinogens^46^. Ecological studies have described geographic patterns in which elevated cancer rates coincide with regions burdened by greater plastic-derived pollution or presumed MNP contamination. These signals, however, should be treated as correlational rather than causal, given the likelihood of residual confounding (including co-occurring industrial emissions, socioeconomic differences, and lifestyle-related determinants) and the frequent imprecision of exposure characterization at the population level^47^. Alarmingly, a rise in both benign and malignant tumors has also been observed in other species, including sea turtles, sea lions, and Tasmanian devils, further supporting the potential oncogenic effects of MNPs pollution^48^.

Evidence from in vitro and in vivo studies suggests that MNPs promote genotoxicity and DNA damage primarily through chronic inflammation and oxidative stress. These processes contribute to the development of a tumor microenvironment favorable for malignant transformation, consistent with inflammation being a key hallmark of cancer^49^. Several groups have tried to determine whether MNP exposure can trigger inflammation and impact immune system activity, measuring the levels of different cytokines involved in the inflammatory process, and in the regulation of innate or adaptive immune responses, such as Interleukin 1 (IL-1), IL-2, IL-6, IL-10, and Tumor necrosis factor alpha (TNF-α)^50,51^. In response to MNPs, an increased secretion of TNF-α, IL-1, and IL-10 was found, suggesting that innate immune cells, such as macrophages and dendritic cells, can recognize MNPs as pathogens and phagocytize them; therefore, MNPs are more likely to trigger innate than adaptive immunity. However, due to their size, MNPs are often difficult to remove and persist within the human body, starting the loop of chronic inflammation through the secretion of pro-inflammatory cytokines such as IL-6 or IL-8, ultimately altering the TME and, in the long term, inducing a shift of the immune system towards an immunosuppressive status^52,53^.

In addition, exposure to micro- and nanoplastics has been linked to elevations in cellular oxidative stress, including increased reactive oxygen species production and disruption of redox homeostasis, which may in turn affect DNA integrity and overall genomic stability. Experimental models also implicate plastic-related additives in gene-expression shifts across pathways involved in inflammation, antioxidant and stress responses, proliferation and apoptosis, and regulation of the cell cycle, including signaling networks such as MAPK, Nrf2, PI3K/Akt, and TGF-β^53^. Further details related to various mechanisms through which MPs cause cancer development are explained in Table 2^49,54–68^.

**Table 2:** Summary of carcinogenic mechanisms associated with micro- and nanoplastics

Mechanism	Key processes & effects	Link to tumor progression	Associated cancers / effects
Pro-inflammatory Response^52–54^	- Trigger immune response (macrophages, neutrophils). - Lead to chronic inflammation due to persistence. - Sustained release of IL-6, TNF-α. - Generation of ROS (reactive oxygen species/RNS.	- Alters tumor microenvironment (TME). - Promotes stromal remodeling, angiogenesis. - Tumor-associated macrophages (TAMs) secrete VEGF. - ROS/RNS cause DNA damage. - Impairs immune surveillance, allowing tumor growth.	Liver Cancer, Lung Cancer, Colon Cancer
Oxidative Stress^55–57^	- Adsorb metal ions (Fe, Zn, Cu) that disrupt redox balance. - Inhibit antioxidant enzymes. - Increase intracellular ROS.	- ROS damage cell membranes, proteins, and DNA. - Causes DNA strand breaks and mutations. - Increased mutation rate drives uncontrolled cell proliferation.	General increased cancer risk
Endocrine Disruption & Hormonal Disruptions^58–60^	- Additives (plasticizers, antioxidants) and adsorbed Endocrine Disrupting Chemicals (EDCs) present in MP leach out. - Phthalates and BPA disrupt estrogen/androgen function. - Bisphenol A mimics thyroid hormones. - EDCs bind to hormone receptors, mimicking or blocking natural hormones.	-Hormone imbalance triggers abnormal cell growth. -Stimulates proliferation in hormone-responsive tissues. -Initiates oxidative stress and inflammation.	Breast Cancer, Prostate Cancer, Ovarian Cancer,
Genotoxicity^49,61,62^	- Direct physical interaction with DNA and cellular components. - ROS-induced DNA damage (strand breaks, fragmentation). - Leaching of toxic additives (plasticizers, stabilizers). - Induction of chromosomal abnormalities and spindle disruptions.	- Causes genetic instability and mutations. - Disruption of cell division leads to aneuploidy (abnormal chromosome number). - Collective DNA damage impairs cell function and increases cancer risk.	General increased cancer risk
Disruption of Cell Signaling Pathways^61,63^	- Endocrine-disrupting chemicals (EDCs) bind to hormone receptors. - Imbalance key pathways: MAPK and PI3K/Akt.-Activate IRE1/XBP1 signaling axis of Endoplasmic Reticulum Stress (ERS) through oxidative stress - Polypropylene activating the mTORC2 and AKT signaling pathways.	- Altered cellular responses lead to developmental problems and cancer. - Expression of apoptotic markers, such as caspase-3 and caspase-12 - Disruption of cell cycle regulation and apoptosis (programmed cell death).	Kidney related Cancer, Breast Cancer
Carcinogenic Properties^49,64^	- Act as carriers for carcinogens (PAHs, heavy metals). - Physical properties (size, shape, charge) aid cellular penetration and tissue accumulation. - Leach carcinogenic additives (e.g., phthalates, flame retardants).	- Direct DNA damage and increased mutation rate. - Promotes cell proliferation and suppresses apoptosis.	Bladder, Reproductive Cancers
Epigenetic Changes^65,66^	- Alterations in DNA methylation(hypo/hypermethylation). - Histone modifications (acetylation, methylation). - Dysregulation of non-coding RNAs (e.g., miRNAs).	- Silencing of tumor suppressor genes or activation of oncogenes. - Altered gene expression in cell growth, apoptosis, and immune response.	Neurotoxicity, Cancer

#### MNPs and prostate cancer risk

Tumor and para-tumor samples were analysed from 22 patients with PCa after radical prostatectomy, providing quantitative and qualitative evidence of the presence of MNPs in tumor tissues through laser direct infrared spectroscopy, scanning electron microscopy, and pyrolysis-gas chromatography-mass spectrometry. MNPs were found in both para-tumor and tumor tissues of human prostate, with higher abundance within tumor tissues. Notably, there was a positive correlation between the abundance of MNPs and the frequency of consumption of take-out food^69^. Additives play a further role in cancer development in relation to MNPs, such as antioxidants and plasticizers, which can disrupt the androgen and estrogen pathways, leading to a higher risk of developing certain cancer subtypes, including PCa^70^. For instance, the urine levels of bisphenols and phthalates levels, measured by an ultra-performance liquid chromatography-tandem mass spectrometry, were higher in 187 patients with PCa compared with 151 healthy controls, especially for some metabolites such as bisphenol A and di-isobutyl phthalate^71^.

#### MNPs and bladder cancer risk

Recently, MNPs have been investigated for their potential role in bladder cancer (BCa) onset. Indeed, the link between chemical exposure to aromatic amines, such as 2-naphthylamine, benzidine, 4-aminobiphenyl, and o-toluidine, and BCa has been widely demonstrated. Other chemicals, such as those used in the dye, textile, rubber, and paint industries, as well as cyclophosphamide and arsenic in drinking water, have also been investigated for their association with a higher risk of developing this tumor.

The presence of MNPs in 10 patients during bladder resection was demonstrated by comparing BCa and control cases through Raman microspectroscopy analysis with hyperspectral imaging performed in the range of 600–1800 cm^−1^. In 13 out of 20 samples, PS-MPNs were detected. The risk of accumulation of MNPs in the bladder was proposed to be a possible factor in the development of cancer, with routine pathological and immunofluorescence tests being unable to detect their presence. The study was unable to answer the question of whether food contamination or ingestion was the cause. However, it was also emphasized that the type of spectroscopy used could potentially underestimate the amount of MNPs, due to the limitation in detecting particles smaller than hundreds of micrometers^72^.

At the American Urology Association (AUA) 2025 congress, a study presented identified primary and secondary MNPs as being associated with a higher risk of BCa in the Ohio population, as observed over 8 years from 2013 to 2021. Application of Bivariate Moran’s I for spatial correlation between waste release and cancer incidence, as well as geographic analysis and Local Indicators of Spatial Association, identified spatial clusters. A higher incidence of BCa was found across some areas with recognized plastic processing waste, associated with MNPs in water and air.

An exhaustive scoping review from 2021 provides the most accurate evidence regarding the presence of MNPs in urine samples from patients with GU tumors, primarily containing PS samples. This agent was able to compete with cisplatin, in cell models, for its cytotoxic and pro-apoptotic effects. Therefore, MNPs represent a threat to the human urinary tract cells by causing toxicity and inflammation, reducing cell survival and viability, and disrupting the MAPK (mitogen-activated protein kinase) pathway, which responds to growth signals and stress^73^.

#### MNPs and RCC risk

Several studies have also investigated the potential relationship between MNPs and RCC, especially for PS and acrylonitrile^74^. There is evidence of MNPs accumulating in kidney tissues, especially in kidney tubular cells (HK2), where they can exert different pro-tumorigenic effects, such as the production of ROS, the disruption of lipidic metabolism, endothelial reticulum-mediated stress damage, inflammation, fibrosis, and autophagy^75^. Furthermore, studies, including those focused on the kidney, suggest that MNPs may exert additive effects with environmental pollutants, potential carcinogens, and high-fat diets, ultimately modifying the tumor microenvironment through activation of the PI3K/Akt/MAPK and IL-17 signaling pathways in endothelial cells, effector CD8⁺ T cells, and proliferating T cells^76^.

Mechanistic research has repeatedly indicated that micro- and nanoplastic exposure can trigger oxidative imbalance, inflammatory signaling, genotoxicity-related outcomes, and disturbances of endocrine function; nevertheless, the body of evidence in humans is still comparatively sparse. Scientific literature is dominated by in vitro work, animal studies, and observational (including ecological) investigations, all of which have important limitations when it comes to drawing causal conclusions^77^. Ecological associations, in particular, can be distorted by factors common in highly industrialized settings, such as concurrent exposure to metals, PAHs, and aromatic amines, as well as by lifestyle and socioeconomic differences, and by substantial uncertainty in measuring external exposure and estimating internal dose. Accordingly, here we focus on reported associations and on biological plausibility, while underscoring that a causal link cannot currently be established. Clarifying whether, and to what extent, MNPs influence cancer risk will require robust prospective cohort studies, more accurate exposure assessment at the individual level, and harmonized analytical protocols to detect and characterize MNPs in human biological matrices.

Establishing a direct causal role for micro- and nanoplastics (MNPs) in genitourinary (GU) cancers would require multiple, mutually reinforcing forms of evidence. From a human research perspective, the strongest support would come from large prospective cohort studies in which MNP exposure is quantified prior to cancer onset. Key requirements would include a demonstrable exposure–response pattern (greater internal exposure corresponding to higher risk) and the persistence of associations after stringent control for major confounders and correlated exposures, such as smoking, occupational contact with aromatic amines, arsenic, metals, PAHs, and socioeconomic and work-related factors. Beyond epidemiologic signals, the case for causality would be markedly strengthened by human-relevant mechanistic data indicating that MNPs are not merely detectable but functionally engaged within GU target tissues. This would entail showing that particles reach and interact with pertinent anatomical and cellular compartments (e.g., the urothelial interface, prostate tissue, and renal tubular regions) and that increasing internal burden aligns with cancer-relevant intermediate endpoints, such as oxidative stress and DNA damage markers, persistent inflammatory activation with microenvironmental remodeling, endocrine-disruption profiles (particularly salient for prostate biology), and signatures of epithelial injury–repair or fibrotic remodeling (notably relevant to kidney and bladder)^78^.

### Micro-nanoplastics and cancer therapy

Engineered micro- and nanoplastics (MNPs) are emerging as versatile platforms for drug delivery and cancer therapy, leveraging their small size, modifiable surfaces, and ability to cross biological barriers. They have been designed to ensure biocompatibility, targeting, and payload release, enabling a wide range of therapeutic applications. Below, we summarize some of their mechanisms of action, but we must keep in mind that environmental MNPs can also behave in a similar, yet uncontrolled, manner, potentially creating pro-tumor effects.

- **Changes in drug disposition (ADME):** Experimental work suggests that micro- and nanoplastics (MNPs) could, in principle, shift the pharmacokinetics of anticancer agents by modifying absorption, tissue distribution, metabolism, and elimination. This would influence both therapeutic activity and toxicity. Proposed explanations include effects at barrier interfaces that shape uptake and systemic availability, and liver-mediated changes that could alter biotransformation capacity, circulating half-life, and drug persistence in blood^79–81^.
- **Transporter- and uptake-related mechanisms linked to resistance phenotypes:** At the cellular level, MNPs have been hypothesized to affect how drugs enter and leave cells, for example by perturbing membrane transport processes and efflux activity that govern intracellular drug concentrations. If such shifts reduce intracellular exposure or promote adaptive stress responses, they could contribute to resistance-like outcomes in experimental settings, although this remains far from established in humans^79–81^.
- **Protein corona formation, opsonization, and immune clearance:** After entering biological fluids, particulate materials can rapidly acquire a coating of adsorbed biomolecules, especially proteins, creating a “corona” that promotes opsonization and recognition by macrophages and other phagocytic cells, thereby shaping uptake and clearance kinetics^82,83^. In theory, repeated environmental exposure to MNPs could modulate innate immune handling of particulates or compete for clearance pathways, potentially influencing responses to other foreign particles, including therapeutic nanocarriers.
- **Tumor-directed delivery** via **controllable particle engineering:** The clinically relevant “nanomedicine” opportunity space largely involves pharmaceutical-grade polymer nanoparticles, not environmentally weathered plastics. These systems are deliberately engineered, through size control, surface chemistry, and optional ligand functionalization, to increase drug accumulation at tumor sites while reducing off-target exposure^79^.
- **Prolonged local exposure and improved penetration for intravesical therapy:** For bladder cancer, polymer carriers are often designed to extend residence time within the bladder and enhance penetration into the urothelium, aiming to raise local drug levels without proportionally increasing systemic burden^84,85^.
- **Immunotherapy support through targeted immunomodulator delivery:** Polymer platforms can be used to package and co-deliver immunomodulators or checkpoint-directed agents to immune cells or tumor-associated compartments, with the goal of strengthening antitumor immune activation and counteracting immunosuppressive features of the tumor microenvironment^86,87^.
- **Imaging and image-guided intervention:** Engineered nanosystems can also function as targeted diagnostic probes, improving sensitivity and spatial precision for detection, prognostic assessment, and intraoperative guidance (e.g., receptor-targeted constructs in prostate cancer)^88,89^.

Across these applications, real-world translation hinges on reproducible manufacturing and characterization, predictable biodistribution and elimination, careful management of immunogenicity (including anti-polymer/anti-PEG issues where relevant), and validation in robust in vivo and clinical studies rather than isolated proof-of-concept demonstrations^90,91^.

## Conclusions

The evidence base on micro- and nanoplastics (MNPs) and genitourinary (GU) oncology is expanding quickly, yet it remains fragmentary and heterogeneous. For now, the most defensible interpretation of “risk” is not demonstrated causality, but a plausible biological rationale. In line with multidisciplinary evaluations of carcinogenic potential, interpretation should emphasize core causal requirements—temporality, dose metrics that reflect realistic internal exposure, and mechanistic models with high human relevance (including organoids and organs-on-chips)—rather than relying on isolated experimental readouts^92^. MNPs have been reported in human matrices connected to the GU system, and experimental work repeatedly identifies cancer-relevant perturbations, including redox imbalance, sustained inflammatory activation, endocrine disruption, and impairment of barrier function. Importantly, early tissue-based observations are emerging in GU oncology: microplastics have been detected in human GU tumor specimens, with higher particle burden and broader polymer profiles in malignant tissue than in matched adjacent non-malignant samples^93^. At the cellular scale, internalized nanoplastics may engage converging stress pathways. They can disrupt autophagic processing and proteostasis, impair mitochondrial function, and elicit endoplasmic reticulum stress. Together, these effects may promote ROS-associated inflammatory signaling. Potential genotoxic sequelae, including DNA damage, have also been discussed in relation to intracellular localization and cell-cycle context, especially in rapidly renewing epithelia^94^. Looking ahead, the principal bottlenecks are methodological and epidemiologic. The field needs harmonized analytical workflows with rigorous contamination prevention to support reproducible detection and quantification, followed by adequately powered prospective studies at the individual level that combine internal MNP measurements with established GU cancer determinants and key co-exposures to evaluate temporality, exposure–response patterns, and organ-specific vulnerability. In parallel, a developing clinical–epidemiological line of inquiry has proposed patient-oriented hypotheses for possible chronic effects, including a provisional “Microplastic Syndrome” construct that is explicitly exploratory and requires longitudinal verification^95^. By contrast, most of the near-term clinical opportunities discussed here arise from engineered polymeric nanomaterials manufactured under controlled specifications. In GU oncology, these platforms can address concrete translational challenges: improving intratumoral delivery, prolonging intravesical residence time, enabling immunomodulation within an immunosuppressive tumor milieu, and strengthening diagnostic accuracy and image-guided interventions. Overall, polymer-based nanoscale materials constitute a true “double-edged sword” in GU oncology, with ambient MNPs as an emerging exposure that warrants rigorous risk characterization and engineered polymeric nanoparticles as regulated diagnostic and therapeutic technologies. On the exposure side, progress depends on moving beyond qualitative detection toward quantitative internal dose measures and, ultimately, causal inference. Achieving this will require coordinated efforts spanning environmental science, analytical chemistry, urology, oncology, immunology, and materials engineering. Only by integrating methodological rigor with mechanistic coherence and translational realism can the field define potential risks linked to pervasive MNP exposure while responsibly leveraging engineered nanomaterials to improve outcomes in urogenital cancers.

## Supplementary information

Transparent Peer Review file

## References

[1] Bermúdez, J. R. & Swarzenski, P. W. A microplastic size classification scheme aligned with universal plankton survey methods. *MethodsX* **8**, 101516 (2021). 34754787 10.1016/j.mex.2021.101516 PMC8563659

[2] Ziani, K. et al. Microplastics: A Real Global Threat for Environment and Food Safety: A State of the Art Review. *Nutrients* **15**, 617 (2023). 36771324 10.3390/nu15030617 PMC9920460

[3] Zhang, K. et al. Understanding plastic degradation and microplastic formation in the environment: A review. *Environ. Pollut.* **274**, 116554 (2021). 33529891 10.1016/j.envpol.2021.116554

[4] Bhowmik, A., Saha, G. & Saha, S. C. Microplastics in Animals: The Silent Invasion. *Pollutants* **4**, 490–497 (2024).

[5] https://www.who.int/publications/who-guidelines, Accessed on, (2026)

[6] Revel, M., Châtel, A. & Mouneyrac, C. Micro (nano) plastics: A threat to human health? *Curr. Opin. Environ. Sci. Health* **1**, 17–23 (2018).

[7] Alimi, O. S., Farner Budarz, J., Hernandez, L. M. & Tufenkji, N. Microplastics and nanoplastics in aquatic environments: aggregation, deposition, and enhanced contaminant transport. *Environ. Sci. Technol.* **52**, 1704–1724 (2018). 29265806 10.1021/acs.est.7b05559

[8] Pletz, M. Ingested microplastics: Do humans eat one credit card per week? *J. Hazard. Mater. Lett.* **3**, 100071 (2022).

[9] Yang, Z. et al. Human Microplastics Exposure and Potential Health Risks to Target Organs by Different Routes: A Review. *Curr. Pollut. Rep.* **9**, 468–485 (2023).

[10] Feng, Y. et al. A systematic review of the impacts of exposure to micro- and nano-plastics on human tissue accumulation and health. *Eco Environ. Health* **2**, 195–207 (2023). 38435355 10.1016/j.eehl.2023.08.002 PMC10902512

[11] Microplastics in drinking-water; ISBN 978-92-4-151619-8 © World Health Organization, (2019).

[12] Dzierżyński, E. et al. Microplastics in the Human Body: Exposure, Detection, and Risk of Carcinogenesis: A State-of-the-Art Review. *Cancers* **16**, 3703 (2024). 39518141 10.3390/cancers16213703 PMC11545399

[13] Al Harraq, A. et al. Effects of Weathering on Microplastic Dispersibility and Pollutant Uptake Capacity. *ACS Environ. Au* **2**, 549–555 (2022). 36411868 10.1021/acsenvironau.2c00036 PMC9673469

[14] Rai, P. K., Sonne, C., Brown, R. J. C., Younis, S. A. & Kim, K. H. Adsorption of environmental contaminants on micro- and nano-scale plastic polymers and the influence of weathering processes on their adsorptive attributes. *J. Hazard. Mater.* **427**, 127903 (2022). 34895806 10.1016/j.jhazmat.2021.127903 PMC9758927

[15] Leslie, H. A. et al. Discovery and quantification of plastic particle pollution in human blood. *Environ. Int* **163**, 107199 (2022). 35367073 10.1016/j.envint.2022.107199

[16] Roslan, N. S. et al. Detection of microplastics in human tissues and organs: A scoping review. *J. Glob. Health* **14**, 04179 (2024). 39175335 10.7189/jogh.14.04179 PMC11342020

[17] Csóka, I., Ismail, R., Jójárt-Laczkovich, O. & Pallagi, E. Regulatory Considerations, Challenges and Risk-based Approach in Nanomedicine Development. *Curr. Med Chem.* **28**, 7461–7476 (2021). 33823761 10.2174/0929867328666210406115529

[18] Su, Y. et al. PLGA-based biodegradable microspheres in drug delivery: recent advances in research and application. *Drug Deliv.* **28**, 1397–1418 (2021). 34184949 10.1080/10717544.2021.1938756 PMC8248937

[19] Ibrahim, M. et al. Polyethylene glycol (PEG): The nature, immunogenicity, and role in the hypersensitivity of PEGylated products. *J. Control Release* **351**, 215–230 (2022). 36165835 10.1016/j.jconrel.2022.09.031

[20] Shi, D. et al. To PEGylate or not to PEGylate: Immunological properties of nanomedicine’s most popular component, polyethylene glycol and its alternatives. *Adv. Drug Deliv. Rev.* **180**, 114079 (2022). 34902516 10.1016/j.addr.2021.114079 PMC8899923

[21] Casella, C., Vadivel, D. & Dondi, D. The Current Situation of the Legislative Gap on Microplastics (MPs) as New Pollutants for the Environment. *Water Air Soil Pollut.* **235**, 778 (2024).

[22] https://echa.europa.eu/hot-topics/microplastics. Accessed on, (2026).

[23] https://eur-lex.europa.eu/eli/reg/2023/2055/oj. Accessed on, (2026).

[24] https://www.unep.org/inc-plastic-pollution/session-5.2). Accessed on, (2026).

[25] Leonard, S. V. L. et al. Microplastics in human blood: Polymer types, concentrations and characterisation using μFTIR. *Environ. Int* **188**, 108751 (2024). 38761430 10.1016/j.envint.2024.108751

[26] Pironti, C. et al. First Evidence of Microplastics in Human Urine, a Preliminary Study of Intake in the Human Body. *Toxics* **11**, 40 (2022). 36668766 10.3390/toxics11010040 PMC9867291

[27] Yun, X. et al. Raman-guided exploration of placental microplastic exposure: Unraveling the polymeric tapestry and assessing developmental implications. *J. Hazard Mater.* **477**, 135271 (2024). 39038382 10.1016/j.jhazmat.2024.135271

[28] Braun, T. et al. Detection of Microplastic in Human Placenta and Meconium in a Clinical Setting. *Pharmaceutics* **13**, 921 (2021). 34206212 10.3390/pharmaceutics13070921 PMC8308544

[29] Ragusa, A. et al. Plasticenta: First evidence of microplastics in human placenta. *Environ. Int* **146**, 106274 (2021). 33395930 10.1016/j.envint.2020.106274

[30] Umurhan, Y., Songsart-Power, M., Limbu, T. B. & Phan, T. Applications of Raman spectroscopy for microplastic detection and characterization: a comprehensive spectral reference. *Environ. Sci. Pollut. Res Int* **32**, 28630–28677 (2025). 41310260 10.1007/s11356-025-37224-3 PMC12714846

[31] Rauert, C. et al. Assessing the Efficacy of Pyrolysis–Gas Chromatography–Mass Spectrometry for Nanoplastic and Microplastic Analysis in Human Blood. *Environ. Sci. Technol.* **59**, 1984–1994 (2025). 39851066 10.1021/acs.est.4c12599 PMC11800385

[32] Primpke, S., Fischer, M., Lorenz, C., Gerdts, G. & Scholz-Böttcher, B. M. Comparison of pyrolysis gas chromatography/mass spectrometry and hyperspectral FTIR imaging spectroscopy for the analysis of microplastics. *Anal. Bioanal. Chem.* **412**, 8283–8298 (2020). 33104827 10.1007/s00216-020-02979-w PMC7680748

[33] Liu, L. et al. Cellular internalization and release of polystyrene microplastics and nanoplastics. *Sci. Total Environ.* **779**, 146523 (2021). 34030247 10.1016/j.scitotenv.2021.146523

[34] González-Caballero, M. C. et al. Internalization and toxicity of polystyrene nanoplastics on inmortalized human neural stem cells. *Chemosphere* **355**, 141815 (2024). 38556182 10.1016/j.chemosphere.2024.141815

[35] Ito, T. et al. Human neurons are susceptible to the internalization of small-sized nanoplastics. *Environ. Toxicol. Pharm.* **118**, 104776 (2025). 10.1016/j.etap.2025.104776 40744315

[36] Lee, D. W. et al. Microplastic particles in human blood and their association with coagulation markers. *Sci. Rep.* **14**, 30419 (2024). 39638849 10.1038/s41598-024-81931-9 PMC11621780

[37] La Porta, E. et al. Microplastics and Kidneys: An Update on the Evidence for Deposition of Plastic Microparticles in Human Organs, Tissues and Fluids and Renal Toxicity Concern. *Int J. Mol. Sci.* **24**, 14391 (2023). 37762695 10.3390/ijms241814391 PMC10531672

[38] Dong C. et al. Microplastic accumulation in endometrial cancer tissues and its metabolic impact. *Environ. Pollut*. **384**, 127005 (2025). 10.1016/j.envpol.2025.127005 40835103

[39] Nawab, A. et al. Human exposure to microplastics: A review on exposure routes and public health impacts. *J. Hazard. Mater. Adv.* **16**, 100487 (2024).

[40] Rahman, A., Sarkar, A., Yadav, O. P., Achari, G. & Slobodnik, J. Potential human health risks due to environmental exposure to nano- and microplastics and knowledge gaps: A scoping review. *Sci. Total Environ.* **757**, 143872 (2021). 33310568 10.1016/j.scitotenv.2020.143872

[41] Lv, W. et al. Underestimated health risks: Dietary restriction magnify the intestinal barrier dysfunction and liver injury in mice induced by polystyrene microplastics. *Sci. Total Environ.* **898**, 165502 (2023). 37451458 10.1016/j.scitotenv.2023.165502

[42] Deng, Y., Zhang, Y., Lemos, B. & Ren, H. Tissue accumulation of microplastics in mice and biomarker responses suggest widespread health risks of exposure. *Sci. Rep.* **7**, 46687 (2017). 28436478 10.1038/srep46687 PMC5402289

[43] Jin, Y., Lu, L., Tu, W., Luo, T. & Fu, Z. Impacts of polystyrene microplastic on the gut barrier, microbiota, and metabolism of mice. *Sci. Total Environ.* **649**, 308–317 (2019). 30176444 10.1016/j.scitotenv.2018.08.353

[44] Xie, X. et al. Exposure to polystyrene microplastics causes reproductive toxicity through oxidative stress and activation of the p38 MAPK signaling pathway. *Ecotoxicol. Environ. Saf.* **190**, 110133 (2020). 31896473 10.1016/j.ecoenv.2019.110133

[45] Masciarelli, E. et al. Microplastics in Agricultural Crops and Their Possible Impact on Farmers’ Health: A Review. *Int. J. Environ. Res. Pub. Health* **22**, 45 (2025). 10.3390/ijerph22010045 PMC11765068 39857498

[46] Baj, J. et al. Derivatives of Plastics as Potential Carcinogenic Factors: The Current State of Knowledge. *Cancers (Basel)* **14**, 4637 (2022). 36230560 10.3390/cancers14194637 PMC9562888

[47] Tang, K. H. D., Li, R., Li, Z. & Wang, D. Health risk of human exposure to microplastics: a review. *Environ. Chem. Lett.* **22**, 1155–1183 (2024).

[48] Erren, T., Zeuss, D., Steffany, F. & Meyer-Rochow, B. Increase of wildlife cancer: an echo of plastic pollution? *Nat. Rev. Cancer* **9**, 842 (2009). 19816457 10.1038/nrc2665-c1

[49] Goswami, S. et al. The alarming link between environmental microplastics and health hazards with special emphasis on cancer. *Life Sci.* **355**, 122937 (2024). 39103046 10.1016/j.lfs.2024.122937

[50] Green, T. R., Fisher, J., Stone, M., Wroblewski, B. M. & Ingham, E. Polyethylene particles of a ‘critical size’ are necessary for the induction of cytokines by macrophages in vitro. *Biomaterials* **19**, 2297–2302 (1998). 9884043 10.1016/s0142-9612(98)00140-9

[51] Hwang, J. et al. Potential toxicity of polystyrene microplastic particles. *Sci. Rep.* **10**, 7391 (2020). 32355311 10.1038/s41598-020-64464-9 PMC7193629

[52] Alijagic, A. et al. Särndahl E. Immunotoxic, genotoxic, and endocrine disrupting impacts of polyamide microplastic particles and chemicals. *Environ. Int.* **183**, 108412 (2024). 38183898 10.1016/j.envint.2023.108412

[53] Vincoff, S. et al. The Known and Unknown: Investigating the Carcinogenic Potential of Plastic Additives. *Environ. Sci. Technol.* **58**, 10445–10457 (2024). 38830620 10.1021/acs.est.3c06840 PMC11191590

[54] Akdis, M. et al. Interleukins (from IL-1 to IL-38), interferons, transforming growth factor β, and TNF-α: Receptors, functions, and roles in diseases. *J. Allergy Clin. Immunol.* **138**, 984–1010 (2016). 27577879 10.1016/j.jaci.2016.06.033

[55] Cassetta, L. & Pollard, J. W. A timeline of tumour-associated macrophage biology. *Nat. Rev. Cancer* **23**, 238–257 (2023). 36792751 10.1038/s41568-022-00547-1

[56] Kidane, D. et al. Interplay between DNA repair and inflammation, and the link to cancer. *Crit. Rev. Biochem. Mol. Biol.* **49**, 116–139 (2014). 24410153 10.3109/10409238.2013.875514 PMC4300235

[57] Wang, X. et al. Recent advances on the effects of microplastics on elements cycling in the environment. *Sci. Total Environ.* **849**, 157884 (2022). 35944635 10.1016/j.scitotenv.2022.157884

[58] Gilardini Montani, M. S. et al. EBV reduces autophagy, intracellular ROS and mitochondria to impair monocyte survival and differentiation. *Autophagy* **15**, 652–667 (2019). 30324853 10.1080/15548627.2018.1536530 PMC6526866

[59] Deng X., Gui Y., Zhao L. The micro(nano)plastics perspective: exploring cancer development and therapy. *Molecular Cancer.* **24**, 30 (2025). 10.1186/s12943-025-02230-z PMC11761189 39856719

[60] Xu, K. et al. Polystyrene microplastics and di-2-ethylhexyl phthalate co-exposure: Implications for female reproductive health. *Environ. Sci. Ecotechnology* **22**, 100471 (2024). 10.1016/j.ese.2024.100471 PMC11363624 39220680

[61] Wang, X. et al. Exploring the relationships between exposure levels of bisphenols and phthalates and prostate cancer occurrence. *J. Hazard. Mater.* **474**, 134736 (2024). 38815394 10.1016/j.jhazmat.2024.134736

[62] Hsu, Y.-L. et al. Obtusifolin suppresses phthalate esters-induced breast cancer bone metastasis by targeting parathyroid hormone-related protein. *J. Agric. food Chem.* **62**, 11933–11940 (2014). 25415928 10.1021/jf5042905

[63] Vlacil, A.-K. et al. Polystyrene microplastic particles induce endothelial activation. *PloS one* **16**, e0260181 (2021). 34788346 10.1371/journal.pone.0260181 PMC8598073

[64] Joseph M. M., Nair J. B., Joseph A. M. Microscopic Menace: Exploring the Link Between Microplastics and Cancer Pathogenesis. *Environ. Sci.*: *Processes Impacts*. 27,1768–1795 (2025). 10.1039/d5em00232j 40497450

[65] Huang, H. et al. Micro/nano plastics in the urinary system: Pathways, mechanisms, and health risks. *Environ. Int*. **193**, 109109 (2024). 10.1016/j.envint.2024.109109 39500122

[66] Kumar, N. et al. Microplastics - A Growing Concern as Carcinogens in Cancer Etiology: Emphasis on Biochemical and Molecular Mechanisms. *Cell Biochem Biophys.* **82**, 3109–3121 (2024). 39031249 10.1007/s12013-024-01436-0

[67] Haleem, N. et al. Microplastics and associated chemicals in drinking water: A review of their occurrence and human health implications. *Sci. Total Environ.* **912**, 169594 (2024). 38154642 10.1016/j.scitotenv.2023.169594

[68] Li, X. et al. PMMA nanoplastics induce gastric epithelial cellular senescence and cGAS-STING-mediated inflammation via ROS overproduction and NHEJ suppression. *Ecotoxicol. Environ. Saf.* **287**, 117284 (2024). 39515200 10.1016/j.ecoenv.2024.117284

[69] Deng, C. et al. Identification and analysis of microplastics in para-tumor and tumor tissues of human prostate. *EBioMedicine* **108**, 105360 (2024). 39341155 10.1016/j.ebiom.2024.105360 PMC11481604

[70] Hsu, Y. L. et al. Obtusifolin suppresses phthalate esters-induced breast cancer bone metastasis by targeting parathyroid hormone-related protein. *J. Agric Food Chem.* **62**, 11933–11940 (2014). 25415928 10.1021/jf5042905

[71] Wang, X. et al. Exploring the relationships between exposure levels of bisphenols and phthalates and prostate cancer occurrence. *J. Hazard Mater.* **474**, 134736 (2024). 38815394 10.1016/j.jhazmat.2024.134736

[72] Krafft, C., Popp, J., Bronsert, P. & Miernik, A. Raman Spectroscopic Imaging of Human Bladder Resectates towards Intraoperative Cancer Assessment]. *Cancers (Basel)* **15**, 2162 (2023). 37046822 10.3390/cancers15072162 PMC10093366

[73] O’Callaghan, L. et al. Plastic induced urinary tract disease and dysfunction: a scoping review. *J. Expo. Sci. Environ. Epidemiol*. 10.1038/s41370-024-00709-3. 10.1038/s41370-024-00709-3 PMC12401723 39217203

[74] Karami, S. et al. Renal cancer risk and occupational exposure to polycyclic aromatic hydrocarbons and plastics. *J. Occup. Environ. Med* **53**, 218–223 (2011). 21270648 10.1097/JOM.0b013e31820a40a3 PMC3065187

[75] Wang, Y. L. et al. The Kidney-Related Effects of Polystyrene Microplastics on Human Kidney Proximal Tubular Epithelial Cells HK-2 and Male C57BL/6 Mice. *Environ. Health Perspect.* **129**, 57003 (2021). 33956507 10.1289/EHP7612 PMC8101928

[76] Zou, H. et al. Microplastics Exacerbate Cadmium-Induced Kidney Injury by Enhancing Oxidative Stress, Autophagy, Apoptosis, and Fibrosis. *Int J. Mol. Sci.* **23**, 14411 (2022). 36430889 10.3390/ijms232214411 PMC9694236

[77] Ali, N. et al. Microplastic and nanoplastic pollution and associated potential disease risks. *Lancet Planet Health* **9**, 101390 (2025). 41421372 10.1016/j.lanplh.2025.101390

[78] O’Callaghan, L. et al. Plastic induced urinary tract disease and dysfunction: a scoping review. *J. Expo. Sci. Environ. Epidemiol.* **35**, 770–784 (2025). 39217203 10.1038/s41370-024-00709-3 PMC12401723

[79] Kim, H., Zaheer, J., Choi, E. J. & Kim, J. S. Enhanced ASGR2 by microplastic exposure leads to resistance to therapy in gastric cancer. *Theranostics* **12**, 3217–3236 (2022). 35547772 10.7150/thno.73226 PMC9065185

[80] Tastet, V., Le Vée, M., Bruyère, A. & Fardel, O. Interactions of human drug transporters with chemical additives present in plastics: Potential consequences for toxicokinetics and health. *Environ. Pollut.* **331**, 121882 (2023). 37236587 10.1016/j.envpol.2023.121882

[81] Ma, X. et al. nanoparticles loaded with 5-fluorouracil for targeted hepatocellular carcinoma imaging and therapy. *Biomaterials* **35**, 2878–2889 (2014). 24411331 10.1016/j.biomaterials.2013.12.045

[82] Zhang, T., Wang, Z., Wu, Y., Zhu, S. & Su, J. Interactions of Micro- and Nanoplastics with Biomolecules: From Public Health to Protein Corona Effect and Beyond. *J. Phys. Chem. B* **129**, 5355–5374 (2025). 40413640 10.1021/acs.jpcb.5c00416

[83] Tran TTm, R. offlerS. R. Interactions between nanoparticle corona proteins and the immune system. *Curr. Opin. Biotechnol.* **84**, 103010 (2023). 37852029 10.1016/j.copbio.2023.103010

[84] Mugabe, C. et al. Paclitaxel incorporated in hydrophobically derivatized hyperbranched polyglycerols for intravesical bladder cancer therapy. *Bju Int.* **103**, 978–986 (2009). 19007363 10.1111/j.1464-410X.2008.08132.x

[85] Tsallas, A., Jackson, J. & Burt, H. The uptake of paclitaxel and docetaxel into ex vivo porcine bladder tissue from polymeric micelle formulations. *Cancer Chemother. Pharmacol.* **68**, 431–444 (2011). 21069339 10.1007/s00280-010-1499-2

[86] Jiang, W. et al. Amplified antitumor efficacy by a targeted drug retention and chemosensitization strategy-based combo nanoagent together with PD-L1 blockade in reversing multidrug resistance. *J. Nanobiotechnol.* **19**, 200 (2021). 10.1186/s12951-021-00947-9 PMC8256488 34225744

[87] Zhang, N. et al. A lipid/PLGA nanocomplex to reshape tumor immune microenvironment for colon cancer therapy. *Regen. Biomater.* **11**, rbae036 (2024). 38628547 10.1093/rb/rbae036 PMC11018539

[88] Gu, F. et al. Aptamer-conjugated multi-walled carbon nanotubes as a new targeted ultrasound contrast agent for the diagnosis of prostate cancer. *J. Nanopart. Res* **20**, 303 (2018). 30524190 10.1007/s11051-018-4407-z PMC6244773

[89] Krishnan, M. A., Yadav, K., Roach, P. & Chelvam, V. A targeted near-infrared nanoprobe for deep-tissue penetration and imaging of prostate cancer. *Biomater. Sci.* **9**, 2295-2312 (2021). 10.1039/d0bm01970d 33554988

[90] Cunha, A., Gaubert, A., Latxague, L. & Dehay, B. PLGA-Based Nanoparticles for Neuroprotective Drug Delivery in Neurodegenerative Diseases. *Pharmaceutics* **13**, 1042 (2021). 34371733 10.3390/pharmaceutics13071042 PMC8309027

[91] D’souza, A. A. & Shegokar, R. Polyethylene glycol (PEG): a versatile polymer for pharmaceutical applications. *Expert Opin. Drug Deliv.* **13**, 1257–1275 (2016). 27116988 10.1080/17425247.2016.1182485

[92] Casella C., Dondi D. & Vadivel D. Do microplastics (MPs) and nanoplastics (NPs) directly contribute to human carcinogenesis? *Environ. Pollut*. **388**, 127343 (2026). 10.1016/j.envpol.2025.127343 41183647

[93] Wang, M. et al. Identification and analysis of microplastics in human penile cancer tissues. *Sci. Total Environ.* **969**, 178815 (2025). 40015124 10.1016/j.scitotenv.2025.178815

[94] Casella, C. & Ballaz, S. J. Genotoxic and neurotoxic potential of intracellular nanoplastics: A review. *J. Appl Toxicol.* **44**, 1657–1678 (2024). 38494651 10.1002/jat.4598

[95] Cornelli, U. et al. Definition of Emerging Microplastic Syndrome Based on Clinical and Epidemiological Evidence: A Narrative Review. *Microplastics* **4**, 93 (2025).

